# A Mixed Modeling Approach to Predict the Effect of Environmental Modification on Species Distributions

**DOI:** 10.1371/journal.pone.0089131

**Published:** 2014-02-26

**Authors:** Francesco Cozzoli, Menno Eelkema, Tjeerd J. Bouma, Tom Ysebaert, Vincent Escaravage, Peter M. J. Herman

**Affiliations:** 1 Spatial Ecology Department, Netherlands Institute of Sea Research, Yerseke, The Netherlands; 2 Hydraulic Engineering Department, Delft University of Technology (TU Delft), Delft, The Netherlands; 3 Institute for Marine Resources and Ecosystem Studies, Wageningen University, Yerseke, The Netherlands; 4 Monitor Taskforce, Netherlands Institute of Sea Research, Yerseke, The Netherlands; Auckland University of Technology, New Zealand

## Abstract

Human infrastructures can modify ecosystems, thereby affecting the occurrence and spatial distribution of organisms, as well as ecosystem functionality. Sustainable development requires the ability to predict responses of species to anthropogenic pressures. We investigated the large scale, long term effect of important human alterations of benthic habitats with an integrated approach combining engineering and ecological modelling. We focused our analysis on the Oosterschelde basin (The Netherlands), which was partially embanked by a storm surge barrier (Oosterscheldekering, 1986). We made use of 1) a prognostic (numerical) environmental (hydrodynamic) model and 2) a novel application of quantile regression to Species Distribution Modeling (SDM) to simulate both the realized and potential (habitat suitability) abundance of four macrozoobenthic species: *Scoloplos armiger*, *Peringia ulvae*, *Cerastoderma edule* and *Lanice conchilega*. The analysis shows that part of the fluctuations in macrozoobenthic biomass stocks during the last decades is related to the effect of the coastal defense infrastructures on the basin morphology and hydrodynamics. The methodological framework we propose is particularly suitable for the analysis of large abundance datasets combined with high-resolution environmental data. Our analysis provides useful information on future changes in ecosystem functionality induced by human activities.

## Introduction

The influence of human activities on Earth's ecosystems has caused changes in global and local scale species distributions [1]. With the recognition of the value of ecosystem services for human communities and the role of biodiversity in delivering these services [2], there is an increasing demand to produce reliable projections of the effects of human interventions on species habitats and distributions. Models able to relate species abundances and environmental conditions (Species Distribution Models, SDMs) are being intensively used in ecological research and conservation planning [3].

Advances in remote sensing and environmental modeling are greatly contributing to the development of SDMs by supplying detailed descriptions of the environment. However, when reliable descriptions of (some) environmental variables are available, several conceptual and analytical issues still need to be investigated in order to increase confidence in the results of SDMs [4], [5]. Species abundances are often the product of different constraints acting at different scales [6]. Even when one (known, measured or modeled) environmental factor is favorable for the species, other (unknown) factors may not, and the species can be absent or limited to a low abundance (Liebig's law of the minimum). As a result, observed species abundances commonly show complex distributional patterns with respect to the known variables. Given the asymmetric distribution of the residuals, such patterns are difficult to interpret with central estimators (*e.g.*, Ordinary Least Square) [7]–[9]. In addition, sampling stochasticity will contribute to variability in the response of the individual sample densities. SDMs usually focus on the ‘true’ responses to the known explanatory variable(s), excluding the variability induced by subsidiary factors. For this reason, they often have been restricted to a partial description of the distribution only, such as modeling of the maximum or binary modeling of presence/absence. This approach expresses species distributions in terms of potential niche or habitat suitability [10]. Habitat suitability fluctuates less in time than realized abundances and it is generally preferred as a reference parameter for spatial management strategies [11]. However, several applications of ecological forecasts require a quantification of the realized abundances rather than just a measure of habitat suitability. There is a need for forecasting models that represent the entire probability distribution of abundance (density, biomass) values at a particular combination of environmental factors [12].

Quantile regression [13], [14] is a statistical technique suitable for the analysis of complex distributional responses [10], [15]–[17]. The method can be used to predict the complete quantile (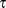) distribution of the response variable 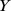 when conditioned by one or more explanatory variables 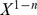: 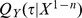. Therefore, regression quantile estimates can be used to construct predictions without specifying how variance heterogeneity is linked to changes in means. Quantile regression models have high performance in explaining the observed variance also in the presence of spatial autocorrelation of environmental variables [8].

Most studies have limited the use of quantile regression to determine the functional relationship between a stressor and the response variable at a limited number of high quantiles (*e.g.*, [18]). Models of the higher quantiles estimate the maximum possible abundance given the known explanatory variables, thus providing estimate of the species potential niche theoretically founded on Liebig's Law. Sub-optimal components of the distribution can be investigated by extending the quantile regression model to the complete range of quantiles [15]. Multiple quantile models have been used to make inferences about the role of the different environmental factors in limiting the different values of the responses [19] or to accurately describe and compare species distributions along single gradients [20].

**Figure 1:**
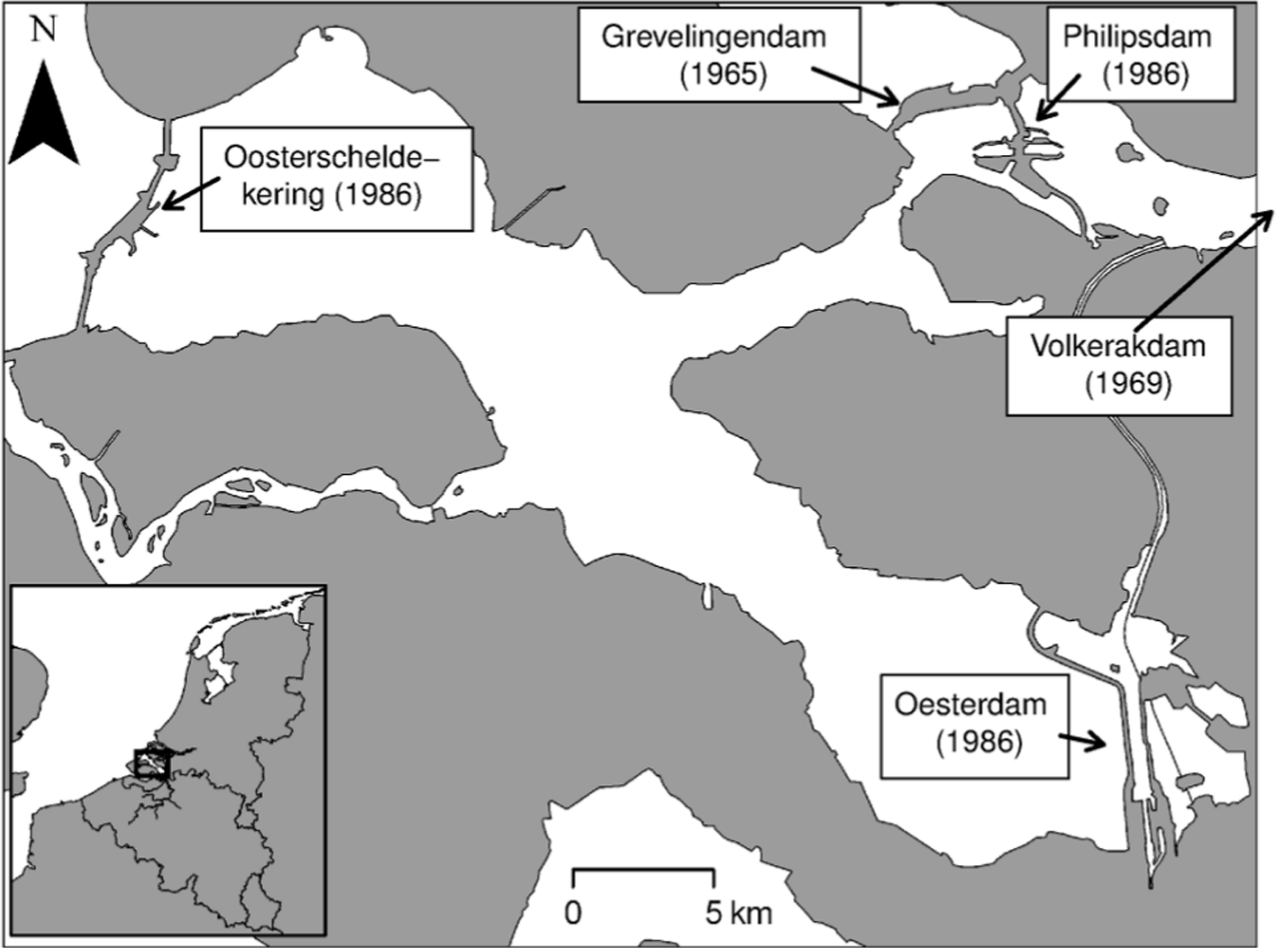
The Oosterschelde basin. In the boxes are reported the name and the realization date of the major dikes.

In this paper we propose a novel integration of numerical hydrodynamic models and SDMs to investigate the response of four common macrozoobenthic species to anthropogenic modifications of their habitat. We chose as study area a temperate coastal embayment in the south - west of The Netherlands: the Oosterschelde (Figure 1). This basin was recently subjected to major human interventions (the realization of coastal defence mega-infrastructures) that deeply affected the basin morpholgy and hydrology [21]. We estimate the consequences on an important component of coastal food webs: the macrozoobenthos [22].

Our study uses a combination of extensive empirical data sets and different types of models. Hydrodynamic variables are known to be among the most important in determining the macrozoobenthic species spatial distribution [23], [24], but they are rarely measured with full spatial coverage, such that they are known for all sample locations. Hydrodynamic and morphodynamic models can fill the gap as they can describe water motion, sediment transport and bed-level changes by numerically solving a coupled set of mathematical equations [25]. Thus, as a first step to investigate the effect of dike building on benthic habitats, we simulated several past, present and future hydrological scenarios of the Oosterschelde by using a numeric hydrodynamic model (DELFT3D). The scenarios are representative of different stages of the recent basin evolution and they can also explore alternative management options, in this case the extreme option of removal of the main storm surge barrier.

Extensive monitoring programmes of macrobenthic fauna have been executed in the Oosterschelde over the past 50 years, with most efforts concentrated in the last 20 years. We combine this information with the results of hydrological models to construct quantile regression SDMs. Upper boundary models emphasize the role of the known variables in determining the species abundance, thus they were used to describe the species potential niche and to produce habitat suitability maps. To express our forecast in realized rather than potential biomass stocks, we account for the complete conditional response distribution forecast by fitting the model on all quantiles. In this way it is possible to reproduce the realistic scattering induced by subsidiary factors with no required assumption about the distributional form (*e.g.*, normal or lognormal) or about the role of the environmental factors (limitation *vs.* facilitation). While the majority of existent studies focus on local/short term disturbances (*e.g.*, bottom disruption, increase turbidity, resuspension of pollutants, look at [26]), the use of prognostic environmental models allow us to investigate the effects of morphological/hydrological alterations on a whole-basin scale and over a time span that is relevant compared to intrinsic morphodynamic time scales.

## Materials and Methods

### Study area

The Oosterschelde (Figure 1) is an enclosed sea arm located in the south of The Netherlands. It was formerly part of a complex delta of the rivers Scheldt, Rhine and Meuse. In 1986, it was partly separated from the North Sea by a storm surge barrier, that can be closed during storm floods. After the realization of the storm surge barrier, the tidal prism (volume of water flowing into or out of an inlet between mean high tide and mean low tide) has been reduced by approximately 30%. Current velocities have declined by 20–40% in the tidal channels and by over 40% around the tidal shoals and salt-marshes [21]. The import of sediment from the coastal sea has been cut off. The availability of suspended sediment for deposition on the flats has decreased considerably, with present suspended particulate matter concentrations being only half those of the pre-barrier situation (on average <20 mg l^−1^) [21]. The decreased tide-induced sediment transport towards the tidal flats relative to the erosion of the flats caused by wind-waves is causing a net erosion of the intertidal area [27]. As a consequence, the channels tend to fill up using sediment eroding from the tidal flats. The erosion mostly affects on the upper intertidal, lowers the mudflats, and is expected to lead to a drastic decrease of the intertidal area ([28], Table 1. The loss of intertidal area is in itself a threat for coastal safety, as the mud and sand flats damp wave energy and protect the dikes behind. It also jeopardizes environmental quality. The Oosterschelde was designated a national park in 2002 and its primary importance as bird feeding area, especially for waders, is recognized in the framework of NATURA2000.

**Table 1:** Areas of the total, subtidal and intertidal surface for the different scenarios.

	1968	1983	1993	2001	2010	2010 (NDW)	2100	2100 (NDW)
Intertidal	171	149	143	147	142	144	65	98
Subtidal	236	234	226	225	227	225	304	271
Total	407	382	370	372	369	369	369	369

Values are in km^2^. NDW (No Delta Works) indicates the results of the scenarios simulated removing the major coastal defense infrastructures.

### Environmental variables

In order to reconstruct the impact of the Delta Works on the macrozoobenthos, we focused on the induced variation in the maximal tidal current velocity (maximal values reached during a full tidal cycle, m sec^−1^) and the inundation time (% of time for which the site is submerged during a full tidal cycle). The sediment composition, traditionally considered as an other important factor for macrozoobenthic species distribution [29], was not considered in this study because it was not possible to compute accurate future scenarios for this variable. The lack of a proper salinity gradient and the limited variation between years in the Oosterschelde [30] make this variable not useful for our purpose.

For this research the Delft3D-Flow model (version 3.55.05.00) is used in two-dimensional depth-averaged mode. The Delft3D-Flow model is discussed in detail in [31]. For application in and around the Oosterschelde, a specific model application has been made, called the KustZuid-model. This model application and its calibration are described in detail in [32]. Historical changes in hydraulic parameters were deduced from seven different model runs, each with a bathymetry from a different year. Sufficient bathymetry data of the basin were available for the years 1968, 1983, 1988, 1993, 2001, 2007 and 2010. The Storm Surge Barrier, Philipsdam, and Oesterdam were excluded from the 1968 and 1983 simulations, and included in the simulations for the years after 1986. Also, the 1968 situation was modeled without the Volkerakdam, so the Volkerak channel is still open. The 2100 scenario was modeled assuming the present trend toward erosion of the intertidal areas/filling of the deepest gullies will linearly continue in future. Additionally, we investigated the effect of the removal of the Delta Works on the 2010 and the 2100 scenarios. Although this is currently not a realistic option for management, these scenarios explore the consequences for the natural morphodynamics (and ecology) of the system. For each of the simulations, the seaward boundary conditions were kept unaltered.

### Biotic variables

#### Benthic dataset

The data used in the present study have been extracted from the Benthic Information System (BIS version 2.01.0) hosted by the NIOZ research center in Yerseke (NL). The BIS database contains about 500000 distribution records about more than 2500 species of all major benthic classes that were collected since 1960 mostly in the Delta region (SW Netherlands). It comprises data from several monitoring projects performed mostly under the authority of Rijkswaterstaat (Dutch Ministry for Public Works and Water Management) in the framework of baseline and impact studies related to the management of the Oosterschelde. A subset of 3342 sampling locations has been selected according to the availability of abiotic data. The 1968 hydrodynamic model was used to extract the environmental conditions for the samples collected between 1962 and 1968. The other scenarios were used to extract the environmental conditions for the samples collected from one year before to one year later than the modeled year (Table 2). When using a dataset combining various monitoring projects with different sampling methods over an extended period of time, metadata have to be carefully checked for different sampling methodologies in order to avoid undue effects of sampling on the observations. The intertidal locations (n = 1372) were mostly sampled by using handcorers pushed 20 to 30 cm in the sediment with a total sampling area between 0.005 and 0.045 m^−2^ (on average 0.019 m^−2^). The subtidal locations (n = 1970) were on some occasions (n = 176) sampled by using Van Veen grabs with a sampling area of 0.1 or 0.2 m^2^ and a penetration depth around 15 cm depending upon the nature of the sediment. In most other cases the subtidal samples consist of subsamples with an average sampling area of 0.023 m^−2^ that were taken by using handcorers pushed 20 to 30 cm in the sediment contained in the bucket of a boxcorer after landing on the ship deck. Whereas most (ca 95%) of the samples have similar characteristics regarding the sediment penetration and the sampling area, the few Van Veen samples stand out due to a ten times larger sampling area and a smaller (1/2) sediment penetration compared to the other samples. Slightly lower density (because of deep living organisms not caught with the Van Veen grab) in the Van Veen samples compared with the handcorer samples have not be taken into account within the present analysis.

**Table 2:** Number of samples included into analysis.

	1962–1968	1985–1989	1992–1994	2000–2002	2006–2008	2008–2010
Intertidal	152	37	541	549	542	149
Subtidal	65	455	138	169	272	273
Total	217	492	679	718	814	422

#### Target response variables

From a preliminary data inspection (Fig. S1), we identified 4 main clusters in the biomass distributions (g m^−2^ Ash Free Dry Weight, AFDW) of the 10 most frequently observed species (relative number of occupied samples). We investigated more in detail the distribution of the most common (or the only) species for each cluster (Table 3):

**Table 3:** Target species characteristics.

Class	Species	Feeding behaviour	Ind. mass (mg AFDW)
Polychaeta	*S. armiger*	Opportunistic deposit feeder	2.6
Gastropoda	*P. ulvae*	Intertidal grazer	0.5
Bivalvia	*C. edule*	Suspension feeder	132
Polychaeta	*L. conchilega*	Selective deposit feeder	15

- *Scoloplos armiger* (bristleworm): intermediate-small motile Polychaeta. It is an opportunistic species, inhabiting a wide range of sedimentary habitats. *S. armiger* is widespread throughout the northern hemisphere and it is the most common species in the Oosterschelde [33].
- *Peringia ulvae* (mudsnail, new name for genus *Hydrobia*): small epibenthic gastropod. This species is mainly distributed in the silty upper intertidal, where it can graze on the benthic diatom film [34]. Despite its small individual body size, it can reach locally a high biomass due to very dense aggregation of individuals.
- *Cerastoderma edule* (common cockle): large shallow burrowing bivalve. It constitutes a predominant portion of the Oosterschelde intertidal biomass [35], [36]. Cockles are a primary food source for avifauna like Oystercatcher and Knot [33].
- *Lanice conchilega* (sand mason): medium-sized sedentary Polychaeta living in tubes that protrude several centimetres from the sediment. Dense aggregates of *L. conchilega* can form sand-reefs that have a relevant influence on the sedimentation [37], [38] and on the ecology of the macrozoobenthic community [39], [40]. The species can be used as a proxy in the management of marine resources and the conservation of marine biodiversity [41], [42].

### Model fitting and validation

Quantile regression [13], [14] is an extension of the linear model that aims at fitting any desired quantile of a response variable distribution to an independent variable. The 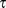-th sample quantile of any random variable Y, 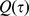, is that value splitting the distribution in a *tau* portion 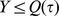 and a 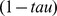 portion 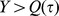. It can be calculated by solving
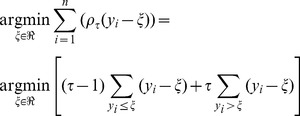
with respect to 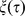. By extension, the linear conditional quantile distribution function 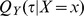 can be estimated by solving
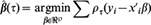



**Figure 2:**
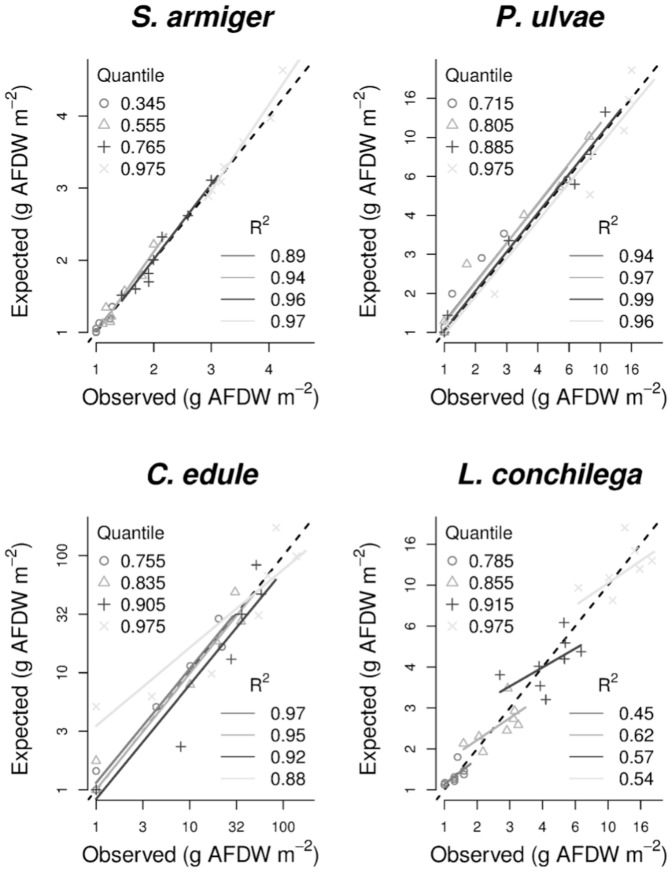
Models validation. Ratio between observed and predicted values. To validate our forecast for each of the modeled quantiles, the whole dataset was sampled with replacement. Due to sampling with replacement, some observations are repeated and others remain unpicked. The model was fitted on the sampled observation (training dataset) and used to predict the unpicked ones (validation dataset). The random sampling-fitting-predicting procedure was iterated 5000 times and repeated for each one of the forecast quantiles. To make predicted (quantiles) and realized values comparable each other, we discretized them in 10 homogeneous classes based on the predicted values. For each of the classes, the correspondent sample quantile of the observed data was calculated. To finally asses the validity of the model, observed and predicted quantiles were plotted against each other and checked for linear correlation. The four quantiles for species showed as examples in the graphs were selected among those predicting occurrence (*e.g.*, up to the 35*^th^* quantile for *S. armiger*, up to the 78*^th^* quantile for *L. conchilega* Table 4). The other quantiles generally follow the same trends. The black broken line represent the 1∶1 ratio.

For each species, the full conditional quantile distribution (from the 0.01 to the 0.99 quantile, with intervals of 0.02) of their biomass (g m^−2^ AFDW) was modeled with respect to the maximal current velocity, the inundation time and their first-degree interaction terms (model selected as the most explicative, Tab. S1 lists AIC scores for different model structures). To validate our forecast for each of the modeled quantiles, the whole dataset was sampled with replacement. Due to sampling with replacement, some observations are repeated and others remain unpicked. The model was fitted on the sampled observations (training dataset) and used to predict the unpicked ones (validation dataset). To obtain a sufficiently large data population, the procedure was iterated 5000 times. The predicted values (expressed as a distributional quantile) were discretized in 10 homogeneous classes, for which the corresponding sample quantile of the validation data was calculated. To finally asses the validity of the model, observed and predicted quantiles were plotted against each other and checked for linear correlation. Examples of four quantiles for each species are shown in Figure 2.

**Figure 3:**
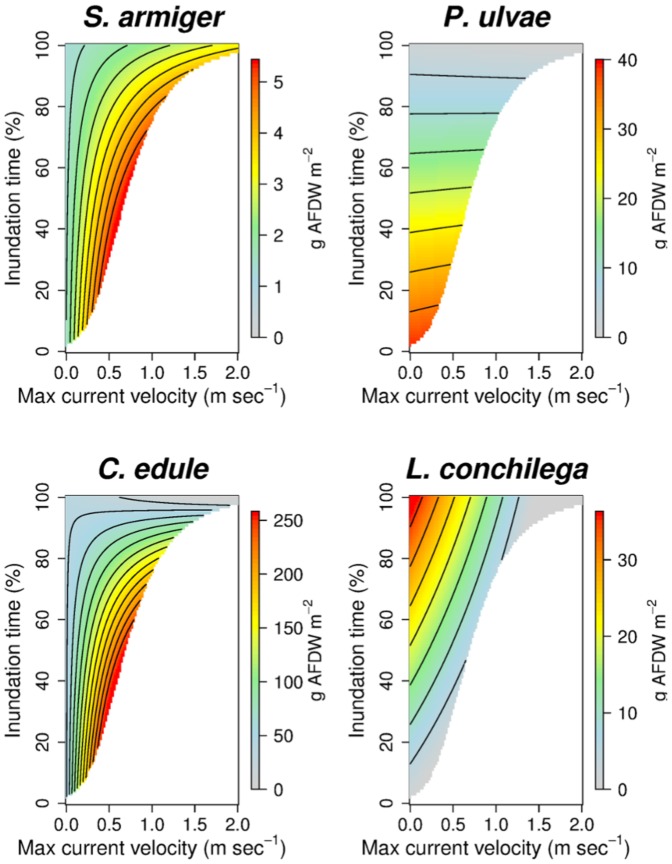
Models of the 0.975*^th^* quantile, response surfaces. Models of the maximal biomass, when extrapolated in the explanatory variable space, give a description of the species potential niche consistent with the Liebig's Law.

**Figure 4:**
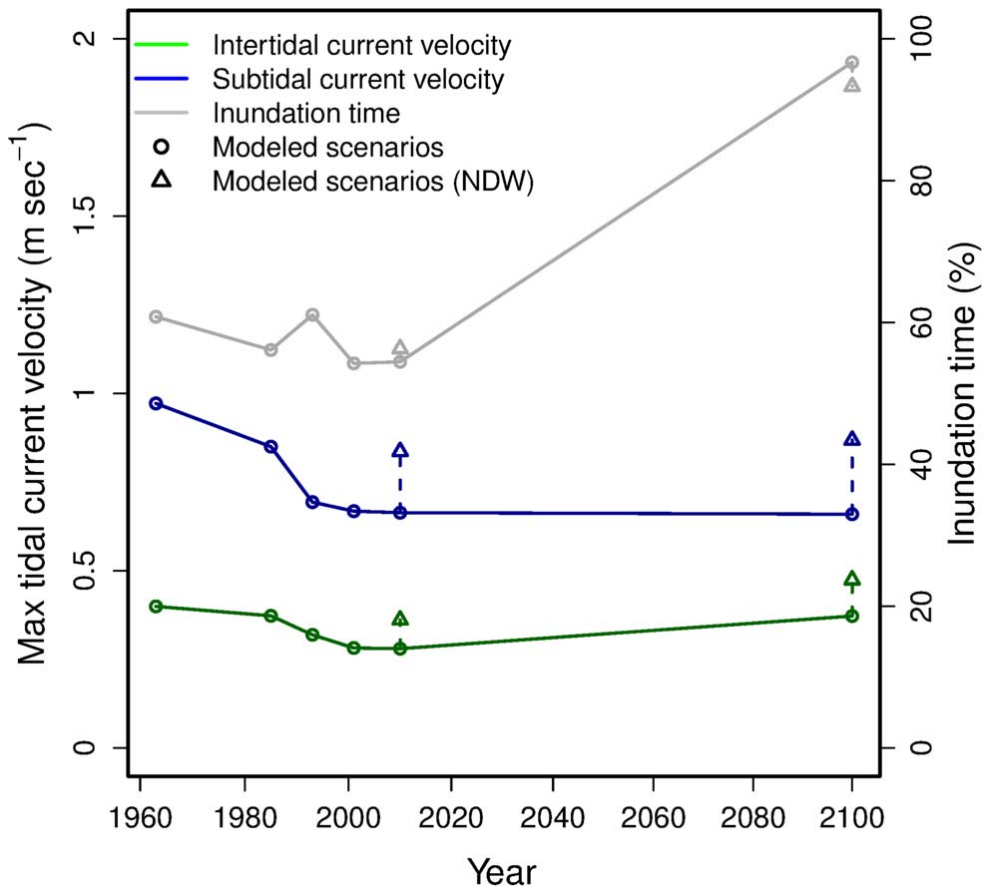
Median values of the explanatory variables on different year-scenarios. Circles represent the median values predicted for the available years-scenarios by the hydrodynamic model. Triangles represent the values predicted for the years 2010 and 2100 removing the Delta Works (NDW).

**Figure 5:**
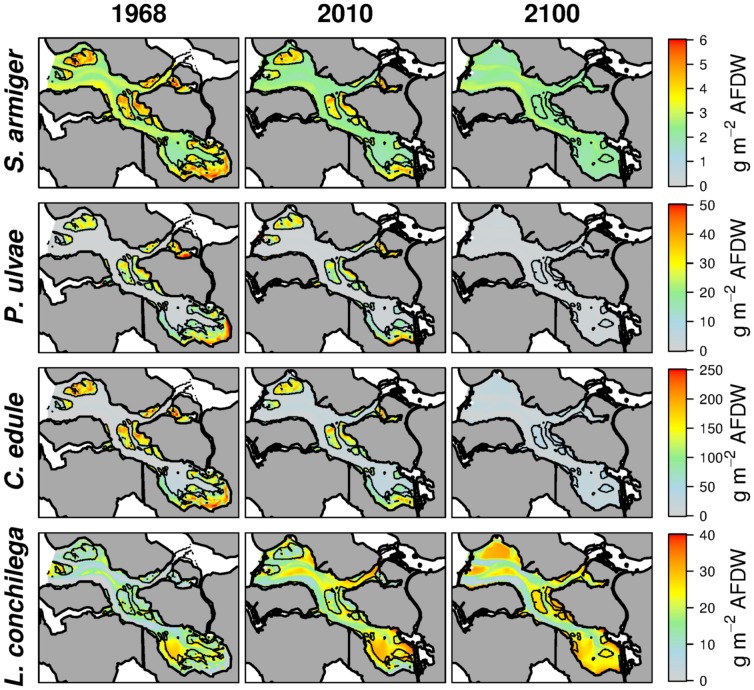
Models of the 0.975*^th^* quantile, habitat suitability. Once extrapolated to realistic scenarios, the response surface shown in 3 are useful to produce clearly interpretable habitat suitability maps. In the figure we show as example the output for the 1968, 2010 and 2100 scenarios.

Given that the maximum can be a fairly volatile statistic due to the influence of outliers [18], we considered a slightly sub-optimal quantile to model the upper boundary of the species responses (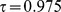, Figure 3). The abiotic scenarios forecasted by the hydrodynamic model (Figure 4) were used to predict maps of potential biomass (habitat suitability) for different years. In the Results section we show the outputs for the years 1968, 2010 and 2100 (Figure 5).

**Figure 6:**
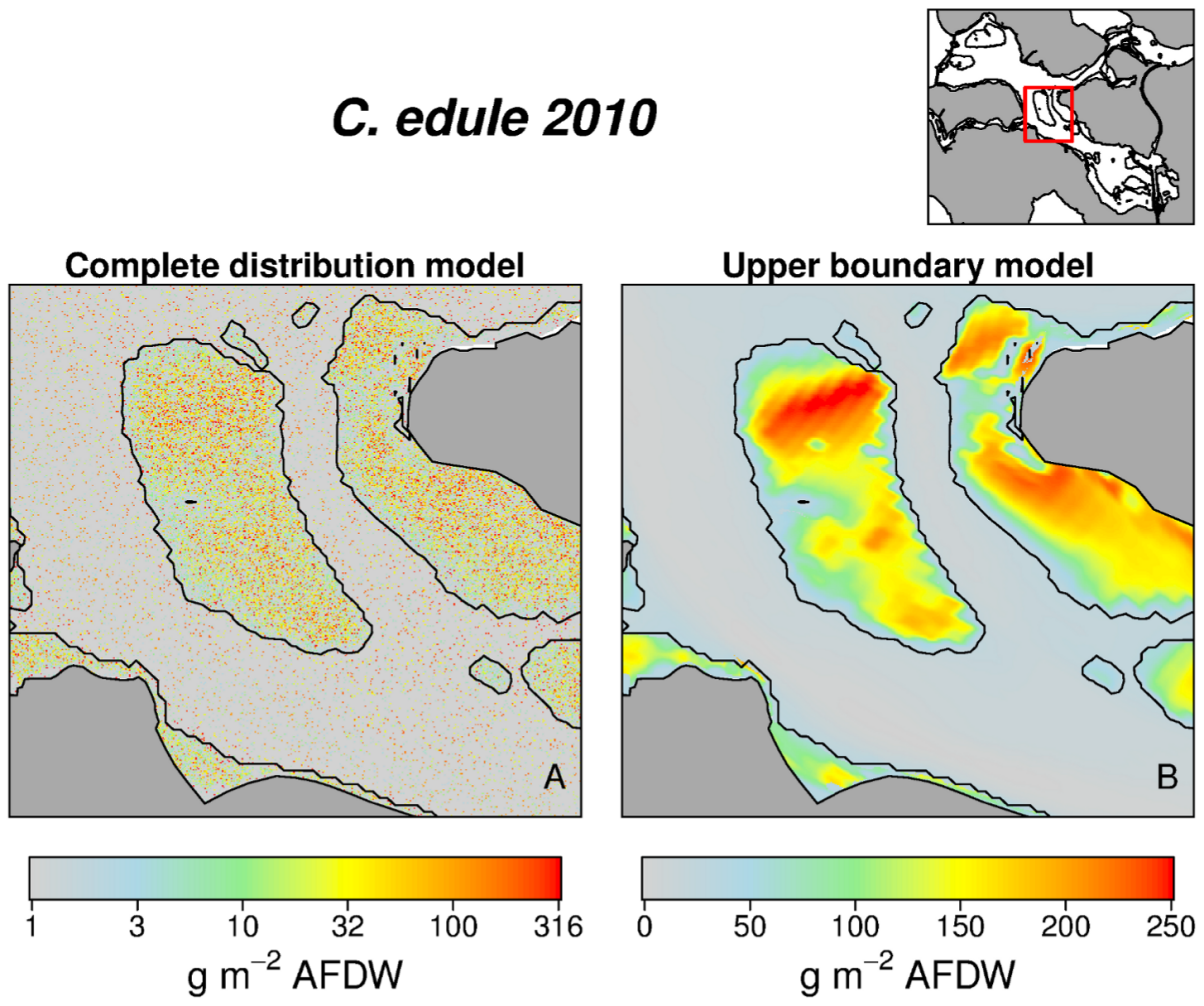
Complete distribution model *vs* Model of the maxima. Example for *C. edule*, year 2010. Map produced by sampling from the complete quantile distribution models (A) are able to represent the realistic scatter around (mainly below) the response surface shown in (B). To help the reader in appreciating the fine mosaic of points in (A) we restricted the map to a smaller portion of the basin and we used a logarithmic scale for plotting the estimated values.

**Figure 7:**
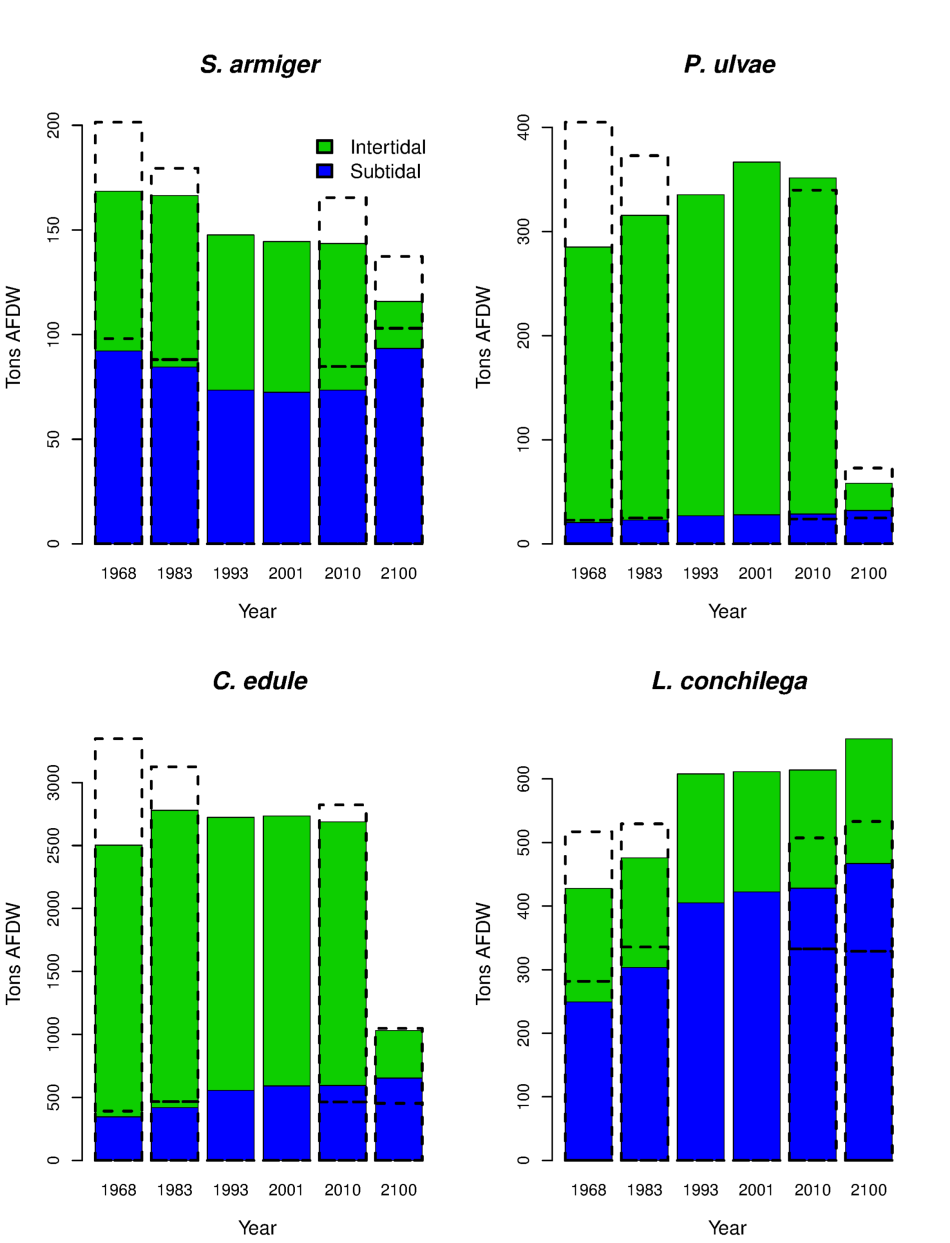
Biomass standing stocks, time series. Colored bar show the intertidal (green) and subtidal (blue) realized biomass stock estimated from the different scenarios for the present extension of the basin. Broken-line bars on the years 1968 and 1983 include the area that was cut-off from the beginning of the Oesterdam works in 1979 (25 km^2^ between 1968 and 1983 and 12 km^2^ between 1983 and 1986). Empty bars on the years 2010 and 2100 show the result of the scenarios simulated removing the Delta Works.

To estimate the total biomass standing stock in each scenario grid cell we randomly sampled a biomass from the forecast conditional distribution (Figure 6). The total biomass stock *T* (Figure 7) were calculated as
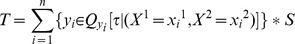
where S is the grid cell surface. Realized stock estimates can slightly differ across different simulations due to stochasticity in the sampling from the conditional quantile distribution. The large number of modeled cells (ca. one million) strongly buffers this uncertainity. In any case, we averaged the outputs of 5 simulations. The error bars are not visible on the scale of the barplots (Figure 7). The inundation time scenarios were used to distinguish between intertidal (inundation time <100%) and subtidal stocks.

All analyses were performed with R [43] mostly using the packges quantreg [44] and raster [45].

## Results

The fitted models (summary tables and graphs in Supporting Information, Tab. S2, S3, S4, S5, Fig. S2, S3, S4, S5) were able to forecast with great accuracy each conditional quantile of the observed distributions (Figure 2). While for *S. armiger*, *P. ulvae* and *C. edule* the ratio between observed and predicted values was very close to 1, the model tended to systematically overestimate the lower values and to underestimate the higher values of the *L. conchilega* realized biomasses (Figure 2). The good match between observed and predicted occurrences (Table 4) indicates that the data scatter below the upper limit is well represented until the threshold for occurrence, even if the predicted values tend to be slightly higher than the observed ones.

**Table 4:** Target species occurrences.

Species	Occurrence (%)					
	Total		Intertidal		Subtidal	
	Observed	Predicted	Observed	Predicted	Observed	Predicted
*S. armiger*	64	65	73	77	58	58
*P. ulvae*	30	34	60	69	9	14
*C. edule*	25	29	55	63	4	9
*L. conchilega*	23	27	17	27	27	27

Observed occurrence are expressed in percentage of occupied samples on the overall dataset. For each modeled scenarios, predicted occurrences were calculated as the percentage of cells for which the model forecast a biomass > =  of the lowest value observed in nature. The predicted occurrence values reported in the table are the average of all the scenarios modeled between 1968 and 2010.

Upper boundary response surfaces (Figure 3) describe the species' potential niche. *P. ulvae* has a clear preference for the sheltered and elevated mudflats. *C. edule* and *S. armiger* share the same optimal habitat in the intertidal zone (intermediate inundation time and moderate hydrodynamic stress), but they diverge for subtidal habitats. While *C. edule* is scarce in permanently inundated sites, *S. armiger* finds a sub-optimal habitat there, especially at strong current velocity. *L. conchilega* preferred subtidal but sheltered habitats (Figure 3).

The analysis of the Oosterschelde abiotic scenarios (Figure 4) shows a decrease in intertidal and subtidal maximal current velocity between 1968 and 1983, due to the realization of the back-barrier dams, and a more consistent drop after 1983 with the realization of the storm surge barrier. Given the ongoing trend in erosion, only a small and shallow portion of the intertidal area will remain in 2100. The removal of the Delta Works could reset the current velocity to the 1968 levels.

Extrapolated on the basis of the abiotic scenarios, upper boundary models provided a clear spatial representation of the species habitat suitability (Figure 5). While *S. armiger* is widely distributed in the basin, the *P. ulvae* and *C. edule* are restricted to the intertidal flats. This implies that the first species, even upon losing its preferential habitat, will be able to cope with the future erosion of the intertidal areas, while the last two will face a drastic decline. High biomasses of *L. conchilega* in 1968 were mostly confined to the eastern part of the basin and to the edge of the mudflats. The reduction of tidal current velocity improved drastically the habitat suitability of the north-east section of the basin for *L. conchilega*. The suitable habitat surface for this species will further increase in future, when the present mudflats will turn to shallow and almost permanently inundated areas.

**Figure 8:**
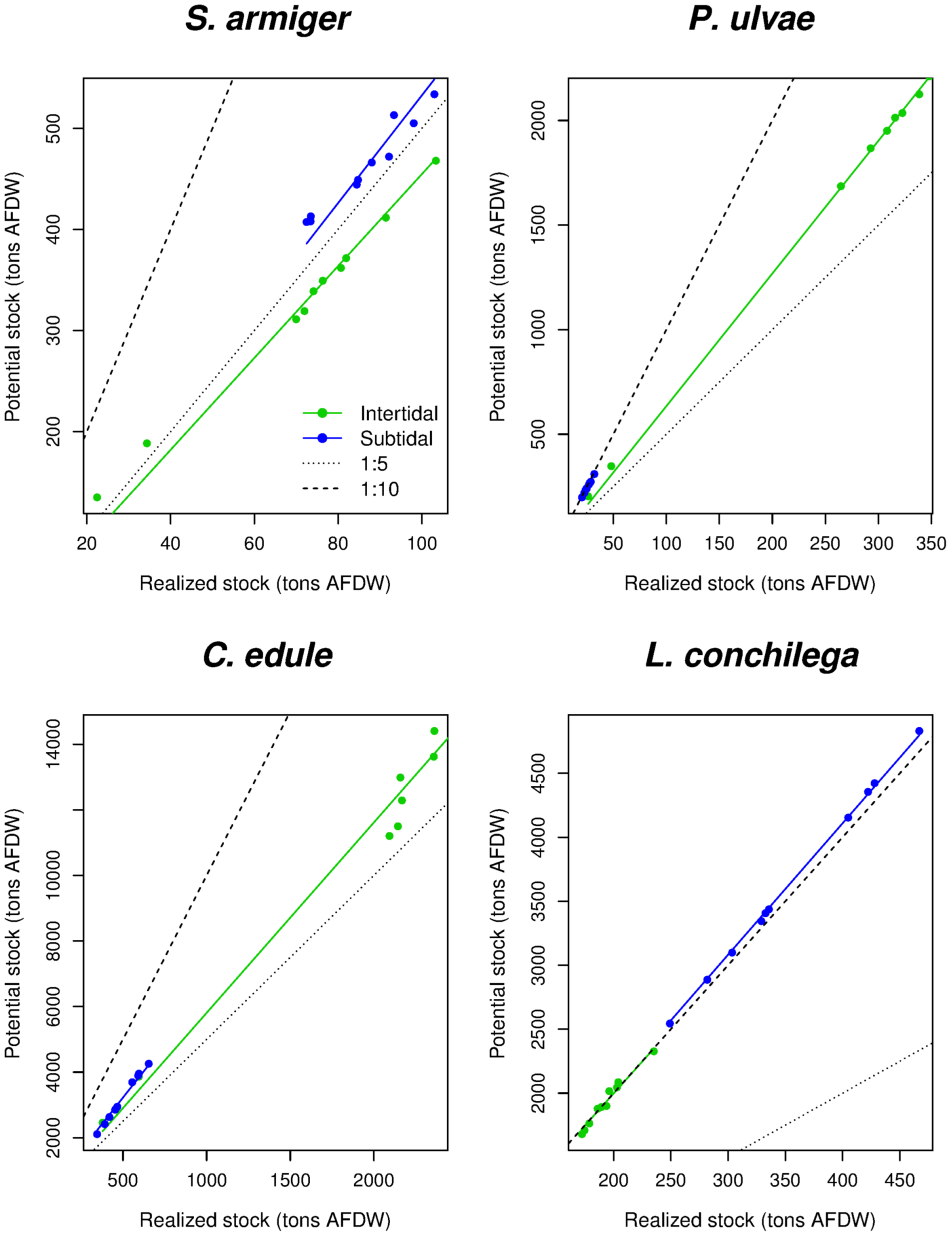
Potential *vs* Realized stocks. The graphs show the ratio between potential (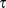 = 0.975) and realized (sampling from the complete cumulative distribution) intertidal (green) and subtidal (blue) biomass stocks estimated for different year/scenarios. The black dotted line represent the 1∶5 ratio. The black broken line represent the 1∶10 ratio.

Maps obtained from sampling the complete conditional quantile distribution (Figure 6 A) show the scatter below (and above, in case of facilitative interaction) the upper boundary surfaces (Figure 6 B). They are more difficult to read than those obtained by modeling just a single quantile, but they represent a more realistic situation. Thus, they can be used to quantify the realized species biomass. The trends in biomass standing stock (Figure 7) show changes between the years 1968–1993 (period of the Delta Works realization) and a relatively stable situation during the last two decades. As shown by 5, the large intertidal area lost between 1979 and 1986 in the eastern part of the basin due to the beginning of the works for the Oesterdam (Figures 1 & Table 1) was able to sustain high biomasses of all the analyzed species. *S. armiger* stock declined after the Delta Works especially in the subtidal habitat. Markedly intertidal species were positively (*P. ulvae*) or fundamentally not (*C. edule*) affected by the changes in the system hydrodynamics (Figure 7), but these species will face a dramatic decline in future due to expected loss of intertidal habitat (Figure 4 & Table 1). For the year 2100 the *C. edule* standing stock is estimated to be ca. 30% (just 10% in the intertidal) of the present situation, while *P. ulvae* will almost disappear from the system. *S. armiger* will be able to partially compensate the decline in the intertidal biomass by establishing in the subtidal habitat. In contrast, *L. conchilega* took advantage from the dampening of current velocities in the channels and increased its biomass by ca. 15% between 1968 and 2001. If the Delta Works are not removed, a further increase in *L. conchilega* is expected in future (Figure 7). Potential biomass standing stocks (from models using 0.975 quantile) are well correlated with the same-year estimations for the realized stocks (Figure 8). The ratio between the realized and the potential stocks varies from ca. 1∶5 (*S. armiger*) to 1∶10 (*L. conchilega*).

## Discussion

A major challenge in SDM is the clarification of the niche concept and the calculation of the influence of each predictor [4]. The methodology we present offers a contribution to this debate. It overcomes the dichotomy between ‘potential’ and ‘realized’ niche, in the sense that our forecast depends on the known environmental gradients but at the same time is fully able to reproduce the variance induced by subsidiary factors. The upper boundary response surfaces offer a synthetic description of the species potential niche (Figure 3). They represent the ‘true’ species response to the known variables, in the sense that they exclude the influence of subsidiary factors on the basis of the Liebig's Law assumptions. This analysis is useful to depict the potentially important areas for the target species (Figure 5). On the other hand, maps obtained by sampling from the full conditional quantile distributions (Figure 6 A) give an image of the biomass values as they could be realistically observed in nature, taking into account the variance induced by subsidiary factors.

### Considerations about the modeling methodology

Models of the full quantile distribution do not require assumptions about the role of the subsidiary factors (*e.g.*, models of the maxima assume that the effects of unmeasured variables will be further limiting rather than facilitative) or about the expected distributional shape (*e.g.*, [46], [47]). While conventional SDM models based on central estimators 1) assume constant error variance, regardless of the value of the predictor variable 2) may fail to distinguish real non-zero changes in zero-inflated distributions, the full quantile distribution model is ‘adaptable’ enough to describe the heterogeneous distributions of the analyzed species. However, phenomena generating endogenous autocorrelation and patchiness at a spatial scale smaller than that of the macrozoobenthos sampling grid (*i.e.*, propagation, aggregation, facilitation, competition) can lead the model to estimate an incorrect ratio between low and high biomass values. This is particularly the case for *L. conchilega* (Figure 2), characterized by a strong aggregational behavior [40], while for the other species the effect is mostly limited to the lower quantiles and can lead to an overestimate of the realized occurrences (Table 4). The strong patchiness in the *L. conchilega* distribution is also evident from the fact that no overlaps are predicted between the values forecast from high and low quantiles (Figure 2).

The close relationship between the potential and the realized estimated stocks (Figure 8) can be explained by interactions and correlations between known and unknown environmental variables, that have the effect to increase the similitude of the responses obtained from different quantiles [8]. The implication is that models of the maxima constitute a good proxy for estimating other components of the distribution, as already shown earlier [48]. However, the degree of scattering beyond the upper boundary (*i.e.*, the realized fraction of the potential stock, Figure 8) is species-specific and it is not possible to derive a generic ‘rule of thumb’ to directly convert potential biomass in realized stocks.

From a practical point of view, this kind of modeling needs a high number of samples to include the complete span of possible combinations between environmental conditions and biomass/abundance. In addition it needs high-resolution environmental layers. In our case we had approximately one milion cells in each of the year scenarios, as the environmental layers were output by the hydrodynamic models. Other examples of similar environmental datasets are satellite images or interpolated surfaces from extensive spatially covering measurements. The use of prognostic environmental models creates the opportunity to extrapolate the results for (hypothetical) past and future conditions, but at the risk of generating error propagation between the environmental model and the SDMs. In the present case, the limited accuracy of the hydrodynamic model in forecasting the environmental conditions at the edge of the mudflats can potentially lead to overestimation of the subtidal biomass of the mainly intertidal species. Moreover, the lower inundation time estimated for the year 1993 (Figure 4) is likely related to lack of resolution in the measured depths close to the shore rather than to effective variations in mudflat elevation or tidal amplitude.

Full quantile distribution models can be used, like in this paper, to quantify the overall effect of environmental changes on realized biomass (Figure 7), and can be useful for ecological applications that cannot rely only on habitat suitability estimations but require accurate information about the realized size of the populations. It should be noted, however, that this approach assumes that the nature of the distributions, and thereby the influence of non-measured subsidiary factors, will remain essentially unchanged. This assumption is difficult to assess in the case of future predictions.

### Comparison with previous estimates (mainly *C. edule*)

The response surfaces forecast by 0.975*^th^* quantile regression are coherent with what is reported in literature for the analyzed species (*e.g.*, [40], [49]). While our representation of the response of *C. edule* to inundation time and current velocity (Figure 3) closely matches with that reported for the Oosterschelde by [36] on the basis of stepwise backward logistic regression, the total biomass standing stock we estimated is approximately 3 times higher than that reported by these authors (27 vs 77 millions kg of wet biomass, assuming a loss of 96% from wet to dry weight [50]). This is related to the fact that logistic regression methods (more in general, occurrence models) are able to give an accurate description of the species presence but definitely underestimate the contribution to the standing stocks of patches with extremely high concentration of individuals.

Compared to previous estimates of *C. edule* standing stocks in the Oosterschelde from large surveys our results show less temporal variability (from 20000 tons AFDW in 1980 to 2000 tons AFDW in 1989 as estimated by [35]). This is related (in addition to large uncertainties and a potentially biased dataset in the analysis of [35]) to the fact that our models average the yearly and seasonal variability by uniformly (“neutrally”) sampling the forecast conditional probability distributions. We made this choice to represent only the amount of variation in standing stocks that can be ascribed to the target explanatory variables. Additional variability is still possible due to trends in large scale subsidiary factors [6] that can restrict the realized output of the forecast distribution to particularly high or low values.

Previous studies applying univariate quantile regression to macrozoobenthic SDM (*i.e.*, [18], [20]), have used non-linear regression techniques (*i.e.*, B-splines transformation of the explanatory variable). This was not necessary in our case: the interactions between the two explanatory variables made the models ‘flexible’ enough to accurately describe the species responses. More tests will be needed to see how general this conclusion is. In any case B-splines transformation could also be used in the multivariate statistical model if needed.

### Temporal trends in the Oosterschelde

The comparison between the upper-boundary response surfaces and the realized biomass stocks allow us to make causal inferences about the fluctuations in species realized biomass across years. *P. ulvae* has maximum habitat suitability in sheltered and elevated sites (Figure 3). The positive trend in *P. ulvae* biomass stocks (Figure 7) from 1968 towards 2010 can be related to the decrease in intertidal tidal currents. For the same reason, species with preferences for intertidal environments with moderate current velocity, like *S. armiger* and *C. edule* (Figure 3) transited through an optimal condition in 1983 (reduction of the current velocity due to the realization of the back-barrier dams) followed by a decline in the following years (further reduction of the tidal currents, mainly due to the realization of the Oosterscheldekering). The effect of the dampening of tidal currents on the biomass of *S. armiger* and *C. edule* diverges in the subtidal environment: negative for *S. armiger* and positive for *C. edule* (Figure 7). Although the decline in the intertidal biomass of *C. edule* was partially compensated by the increase in the subtidal zone, the overall outcome suggests a decrease in the *C. edule* potential as food resource for the avifauna (especially waders like Oystercatcher).

*L. conchilega* prefers subtidal sites with weak currents (Figure 3). It was positively influenced (Figure 7) by the dampening of the tidal current velocity (Figure 4). In particular the realization of the Philipsdam and of the Volkerakdam induced a net increase in habitat suitability in the northern branch of the basin (Figure 5).

While in the last two decades the situation was rather stable for all the species (Figure 7), the future shrinking of the intertidal flats (Table 1, Figure 4) will induce a severe collapse of the standing stocks of *C. edule* and *P. ulvae*. Conversely, *L. conchilega* will reach the highest abundance in 2100, expanding its distribution on the shallow subtidal areas that will take the place of the present-time mudflats (Figure 5). *L. conchilega* is a powerful ecosystem engineer [51], [52], able to stabilize the sediment and increase sedimentation [37], [38]. Therefore, colonizing of the lowering mudflat, *L. conchilega* can reduce the expected intertidal erosion. The decline in *C. edule* and *P. ulvae* biomass (both the species are believed to increase sediment erosion, either directly [53], [54] or by disrupting the benthic diatoms film [55], [56]) could have as well the effect to slow down the loss of intertidal areas.

At the present time, the removal of the Delta Works *per se* would not have an important positive effect on *C. edule* and *P. ulvae* (Figure 7), but it can be useful to slow down the erosion of the intertidal habitat. However, given that the realization of the Delta Works just amplified the pre-existent trend for sediment export [32], some loss of habitat is always expected in the future. Once the erosion process will be very advanced (year 2100), the wider tidal range consequent to the removal of the dikes could increase the intertidal surface (Table 1), helping in preserving a (small) part of *C. edule* and *P. ulvae* habitat. On the other hand, the removal of the coastal defense system would reduce the biomass stock of *L. conchilega* to just a slightly higher value than in the pre-Delta Works state. The only species that could substantially benefit from the removal of the Delta Works is *S. armiger* (Figure 7), that usually is not considered as a target for management strategies.

Retracing the past evolution of the Oosterschelde has given us the opportunity to build and validate models predicting macrozoobenthic community responses to environmental conditions as well as the anthropogenic modification of those conditions. However, in considering these forecasts, it should not be forgotten that they assume that the influence of non-measured subsidiary factors will remain constant through time. This assumption is difficult to assess in the case of future predictions.

At the time of constructing the storm surge barrier, it was already foreseen that tidal currents in the Oosterschelde would decrease in intensity (Figure 4) and that this would lead to enhanced erosion of intertidal flats [57]. This increased erosion is effectively observed [58], and different measures are taken to mitigate the effect. After a first trial, it is planned to regularly use dredge spoil dumped onto the tidal flats as nourishment [28]. Softer defense measures include artificially constructed oyster banks [59] and saltmarsh restoration [60]. The emphasis placed on these measures is related to the conservation goals, as legally fixed *e.g.*, in Natura2000 objectives.

What was not foreseen at the time of embankement, was the striking improvement in quality of the subtidal benthic habitat (Figures 5 & 7). The dampening of current stress allowed a vast portion of the subtidal Oosterschelde to be colonized by large macrozoobenthic organisms, which were confined to the inner and sheltered part of the estuary before the embankements. This change in habitats has created opportunities for touristic (diving) activities, in particular in combination with the increased transparency of the water. The evolution demonstrates that natural values of the original system, such as intertidal productivity and food provision for birds, are intrinsically incompatible with the management option for coastal safety that was chosen, but that other natural values such as subtidal benthic habitat quality do have the potential to be compatible with this option. A public debate is needed on whether nature conservation goals can and should be brought closer in line with other management objectives, or whether natural values should be constraining other management options.

## Conclusion

The methodology we presented allows a realistic representation of species abundances on the basis of known environmental variables. The estimation of realized abundance rather than just habitat suitability revealed extra information on the sensitivity of species to environmental factors [8], [15], [19] and on their population dynamics and energetics [61], [62]. Quantile regression requires limited assumptions about the expected distributional shape and the interactions between explanatory variables. Therefore, it can be applied to a broad range of environments and organisms. The integration between numerical and statistical models is a versatile method for summarizing and simulating the response of species to environmental gradients. This study emphasize the importance of large and long term environmental monitoring programs, as they provide an useful source of information to forecast future ecosystem developments.

Ecological forecast must be included into dynamic infrastructure design to maintain operational efficiency and reduce the ecological impacts [63]. Model extrapolations of the biological and physical environment are a fundamental step to explicitly integrate nature into infrastructure development and to forecast the future availability of ecosystem services [64]. We showed that the realization of surge barriers has mixed and depth-dependent responses that also include improvement of environmental quality. Under this perspective, the analysis of Oosterschelde basins is a precious source of information to understand (and communicate) the future ecological consequences of global trends in human coastal development. The proposed framework can be applied to plan human interventions in a way to minimize their impact or, more optimistically, to maximize their benefits for target species.

## Supporting Information

Figure S1 **Cluster analysis on biomass distributions (g AFDW m^−2^) of the 10 most common species in the Oosterschelde between 1963 and 2010.**
(EPS)

Figure S2 **Variations of the coefficients with respect to modeled quantile for** ***S. armiger*** **.** vel = current velocity, em = emersion time.
(EPS)

Figure S3 **Variations of the coefficients with respect to modeled quantile for** ***P. ulvae*** **.** vel = current velocity, em = emersion time.
(EPS)

Figure S4 **Variations of the coefficients with respect to modeled quantile for** ***C. edule*** **.** vel = current velocity, em = emersion time.
(EPS)

Figure S5 **Variations of the coefficients with respect to modeled quantile for** ***L. conchilega*** **.** vel = current velocity, em = emersion time.
(EPS)

Table S1 AIC scores for different models (average of the AIC scores of all the fitted quantiles).
(TEX)

Table S2 *S. armiger*, summary of the 0.975 quantile (upper boundary) model.
(TEX)

Table S3 *P. ulvae*, summary of the 0.975 quantile (upper boundary) model.
(TEX)

Table S4 *C. edule*, summary of the 0.975 quantile (upper boundary) model.
(TEX)

Table S5 *L. conchilega*, summary of the 0.975 quantile (upper boundary) model.
(TEX)

## References

[1] Parmesan C (2006) Ecological and evolutionary responses to recent climate change. Annual Review of Ecology Evolution and Systematics 37: 637–669.

[2] Costanza R, d'Arge R, deGroot R, Farber S, Grasso M, et al (1997) The value of the world's ecosystem services and natural capital. Nature 387: 253–260.

[3] Syfert MM, Smith MJ, Coomes DA (2013) The Effects of Sampling Bias and Model Complexity on the Predictive Performance of MaxEnt Species Distribution Models. PLOS ONE 8. 10.1371/journal.pone.0055158 PMC3573023 23457462

[4] Araujo M, Guisan A (2006) Five (or so) challenges for species distribution modelling. Journal of Biogeography 33: 1677–1688.

[5] Kamino LHY, Stehmann JR, Amaral S, De Marco P Jr, Rangel TF, et al (2012) Challenges and perspectives for species distribution modelling in the neotropics. Biology Letters 8: 324–326. 22031720 10.1098/rsbl.2011.0942 PMC3367727

[6] Thrush SF, Hewitt JE, Herman PMJ (2005) Multi-scale analysis of species-environment relation-ships. Marine Ecology Progress Series 302: 13–26.

[7] Thomson JD, Weiblen G, Thomson BA, Alfaro S, Legendre P (1996) Untangling multiple factors in spatial distributions: Lilies, gophers, and rocks. Ecology 77: 1698–1715.

[8] Cade BS, Noon BR, Flather CH (2005) Quantile regression reveals hidden bias and uncertainty in habitat models. Ecology 86: 786–800.

[9] Blackburn TM, Lawton JH, Perry NJ (1992) A method of estimating the slope of upper-bounds plots of body size and abundance in natural animal assemblages. Oikos 65: 107–112.

[10] Franklin J (2009) Mapping Species Distributions - Spatial Inference and Prediction. Ecology, Biodiversity and Conservation. Cambridge University Press.

[11] Degraer S, Verfaillie E, Willems W, Adriaens E, Vincx M, et al (2008) Habitat suitability modelling as a mapping tool for macrobenthic communities: An example from the Belgian part of the North Sea. Continental Shealf Research 28: 369–379.

[12] Thrush SF, Hewitt JE, Norkko A, Nicholls PE, Funnell GA, et al (2003) Habitat change in estuaries: predicting broad-scale responses of intertidal macrofauna to sediment mud content. Marine Ecology Progress Series 263: 101–112.

[13] Koenker R, Basset G (1978) Regression quantiles. Econometrica 46: 33–50.

[14] Koenker R, Hallock K (2001) Quantile regression. Journal of Economic Perspectives 15: 143–156.

[15] Cade BS, Noon BR (2003) A gentle introduction to quantile regression for ecologists. Frontiers in Ecology and the Environment 1: 412–420.

[16] Austin MP (2007) Species distribution models and ecological theory: A critical assessment and some possible new approaches. Ecological Modelling 200: 1–19.

[17] Downes BJ (2010) Back to the future: little-used tools and principles of scientific inference can help disentangle effects of multiple stressors on freshwater ecosystems. Freshwater Biology 55: 60–79.

[18] Anderson MJ (2008) Animal-sediment relationships re-visited: Characterising species distributions along an environmental gradient using canonical analysis and quantile regression splines. Journal of Experimental Marine Biology and Ecology 366: 16–27.

[19] Schmidt T, Clements W, Cade B (2012) Estimating risks to aquatic life using quantile regression. Freshwater Science 31: 709–723.

[20] Cozzoli F, Bouma TJ, Ysebaert T, Herman P (2013) An application of non-linear quantile regres-sion to macrozoobenthic species distribution modelling: comparing two contrasting basin. Marine Ecology Progress Series 475: 119–133.

[21] Louters T, van den Berg JH, Mulder JPM (1998) Geomorphological changes of the oosterschelde tidal system during and after the implementation of the delta project. Journal of Coastal Research 14: 1134–1151.

[22] Borja A, Barbone E, Basset A, Borgersen G, Brkljacic M, et al (2011) Response of single benthic metrics and multi-metric methods to anthropogenic pressure gradients, in five distinct European coastal and transitional ecosystems. Marine Pollution Bulletin 62: 499–513. 21215975 10.1016/j.marpolbul.2010.12.009

[23] Allen JR (1985) Field measurement of longshore sediment transport sandy hook, New Jersey, USA. Journal of Coastal Research 1: 231–240.

[24] Snelgrove PVR, Butman CA (1994) Animal-sediment relationships revisited: cause versus effect. Oceanography and Oceanography and Marine Biology-An Annual Review 32: 111–177.

[25] De Vriend HJ, Zyserman J, Nicholson J, Roelvink JA, Pchon P, et al (1993) Medium-term 2dh coastal area modelling. Coastal Engineering 21: 193–224.

[26] Short F, Wyllie-Echeverria S (1996) Natural and human-induced disturbance of seagrasses. Envi-ronmental Conservation 23: 17–27.

[27] De Vriend HJ, Louters T, Berben F, Steijn RC (1989) Hybrid prediction of a sandy shoal evolution in a mesotidal estuary. In: Falconer RA, Goodwin P, Matthew RGS, editors, Hydraulic and Envi-ronmental Modelling of Coastal, Estuarine and River Waters. International Conference Bradford, England, volume 14, pp. 145–156.

[28] Jongeling T (2007) Zandhonger Oosterschelde: maatregelen ter vergroting van doorstroomca-paciteit en zanddoorvoer stormvloedkering Oosterschelde. Technical report, Deltares.

[29] Gray JS (1974) Animal-sediment relationships. Oceanography and Marine Biology Annual Review 12: 223–261.

[30] Haas H (2008) Effecten van een zout volkerak-zoommeer op de ooster- en de westerschelde. Tech-nical report, Rijkwaterstaat.

[31] Lesser GR, Roelvink JA, van Kester JATM, Stelling GS (2004) Development and validation of a three-dimensional morphological model. Coastal Engineering 51: 883–915.

[32] Eelkema M, Wang ZB, Stive MFJ (2012) Impact of back-barrier dams on the development of the ebb-tidal delta of the eastern scheldt. Journal of Coastal Research 28: 1591–1605.

[33] Holtmann S, Groenewold A, Schrader K, Asjes J, Craeymeersch J, et al. (1996) Atlas of the zooben-thos of the Dutch continental shelf. Ministry of Transport, Public Works and Water Management: Rijswijk.

[34] Fenchel T, Kofoed HL, Lappalainen A (1975) Particle size-selection of two deposit feeders: the amphipod *corophium volutator* and the prosobranch *hydrobia ulvae* . Marine Biology 30: 119–128.

[35] Coosen J, Twisk F, van der Tol MWM, Lambeck RHD, van Stralen MR, et al (1994) Variability in stock assessment of cockles (*cerastoderma edule*, l.) in the oosterschelde (in 1980–1990), in relation to environmental factors. Hydrobiologia 283: 381–395.

[36] Kater B, Geurts van Kessel A, Baars J (2006) Distribution of cockles *cerastoderma edule* in the eastern scheldt: habitat mapping with abiotic variables. Marine Ecology Progress Series 318: 221–227.

[37] Carey DA (1987) Sedimentological effects and palaeoecological implications of the tube-building polychaete *lanice conchilega* . Sedimentology 34: 46–66.

[38] Borsje BW, de Vries MB, Hulscher SJMH, de Boer GJ (2008) Modeling large-scale cohesive sedi-ment transport affected by small-scale biological activity. Estuarine, Coastal and Shelf Science 78: 468–480.

[39] Zuhlke R (2001) Polychaete tubes create ephemeral community patterns: *Lanice conchilega* (pallas, 1766) associations studied over six years. Journal of Sea Research 46: 261–272.

[40] Degraer S, Wittoeck J, Appeltans W, Cooreman K, Deprez T, et al (2006) The macrobenthos atlas of the Belgian part of the North Sea. Belgian Science Policy

[41] Rabaut M, Guilini K, Van Hoey G, Magda V, Degraer S (2007) A bio-engineered soft-bottom envi-ronment: The impact of *lanice conchilega* on the benthic species-specific densities and community structure. Estuarine Coastal and Shelf Science 75: 525–536.

[42] Rabaut M, Vincx M, Degraer S (2009) Do *Lanice conchilega* (sandmason) aggregations classify as reefs? Quantifying habitat modifying effects. Helgoland Marine Research 63: 37–46.

[43] R Development Core Team (2011) R: A Language and Environment for Statistical Computing. R Foundation for Statistical Computing, Vienna, Austria. ISBN 3-900051-07-0.

[44] Koenker R (2013) quantreg: Quantile Regression. R package version 4.98.

[45] Hijmans RJ, van Etten J, Mattiuzzi M, Sumner M, Greenberg JA, et al.(2013) raster: Geographic data analysis and modeling. R package version 2.1-37.

[46] He F, Gaston KJ (2007) Estimating abundance from occurrence: An underdetermined problem. American Naturalist 170: 655–659. 10.1086/521340 29522353

[47] Wenger SJ, Freeman MC (2008) Estimating spe occurrence, abundance and detection proability using zero-inated distributions. Ecology 89: 2953–2959. 18959332 10.1890/07-1127.1

[48] VanDerWal J, Shoo LP, Johnson CM, Williams SE (2009) Abundance and the environmental niche: Environmental suitability estimated from niche models predicts the upper limit of local abundance. The American Naturalist 174: 282–291. 10.1086/600087 19519279

[49] Wolff WJ (1983) Estuarine benthos. In: Ketchum BH, editor, Ecosystems of the world: estuaries and enclosed seas, Elsevier, New York, NY. pp. 121–132.

[50] Ricciardi A, Bourget E (1998) Weight-to-weight conversion factors for marine benthic macroinver-tebrates. Marine Ecology Progress Series 163: 246–251.

[51] Jones CG, Lawton JH, Shachak M (1994) Organisms as ecosystem engineers. Oikos 69: 373–386.

[52] Jones CG, Lawton JH, Shachak M (1997) Positive and negative effects of organisms as physical ecosystem engineers. Ecology 78: 1946–1957.

[53] Orvain F, Sauriau P, Bacher C, Prineau M (2006) The inuence of sediment cohesiveness on bioturbation effects due to *Hydrobia ulvae* on the initial erosion of intertidal sediments: A study combining flume and model approaches. Journal of Sea Research 55: 54–73.

[54] Ciutat A, Widdows J, Pope ND (2007) Effect of ýtCerastoderma edule density on near-bed hydro-dynamics and stability of cohesive muddy sediments. Journal of Experimental Marine Biology and Ecology 346: 114–126.

[55] Ubertini M, Lefebvre S, Gangnery A, Grangere K, Le Gendre R, et al (2012) Spatial Variability of Benthic-Pelagic Coupling in an Estuary Ecosystem: Consequences for Microphytobenthos Resuspension Phenomenon. PLOS ONE 7. 10.1371/journal.pone.0044155 PMC3430628 22952910

[56] Montserrat F, Van Colen C, Degraer S, Ysebaert T, Herman PMJ (2008) Benthic community-mediated sediment dynamics. Marine Ecology Progress Series 372: 43–59.

[57] Van den Berg J (1982) Migration of large-scale bedforms and preservation of crossbedded sets in highly accretional parts of tidal channles in the Oosterschelde, SW Netherlands. Geologie en Mijnbouw 61: 253–263.

[58] Bijker W (2002) The Oosterschelde storm surge barrier - A test case for Dutch water technology, management, and politics. Technology and Culture 43: 569–584.

[59] Ysebaert T, Walles B, Dorsch C, Dijkstra J, Troost K, et al (2012) Ecodynamic solutions for the protection of intertidal habitats: the use of oyster reefs. Journal of Shellfish Research 31: 362.

[60] Suykerbuyk W, Bouma TJ, van der Heide T, Faust C, Govers LL, et al (2012) Suppressing antago-nistic bioengineering feedbacks doubles restoration success. Ecological Applications 22: 1224–1231. 22827130 10.1890/11-1625.1

[61] Blackburn T, Brown V, Doube B, Greenwood J, Lawton J, et al (1993) The relationship between abundance and body-size in natural animal assembalges. Journal of Animal Ecology 62: 519–528.

[62] Marquet P, Navarrete S, Castilla J (1995) Body-size, population-density, and the energetic equiv-alence rule. Journal of Animal Ecology 64: 325–332.

[63] Matthews JH, Wickel BAJ, Freeman S (2011) Converging Currents in Climate-Relevant Conser-vation: Water, Infrastructure, and Institutions. PLOS Biology 9. 10.1371/journal.pbio.1001159 PMC3167792 21909243

[64] Chan K, Shaw M, Cameron R, Underwood E, Daily G (2006) Conservation planning for ecosystem services. PLOS Biology 4: 2138–2152. 10.1371/journal.pbio.0040379 PMC1629036 17076586

